# Context, mechanisms and outcomes of integrated care for diabetes mellitus type 2: a systematic review

**DOI:** 10.1186/s12913-015-1231-3

**Published:** 2016-01-15

**Authors:** Loraine Busetto, Katrien Ger Luijkx, Arianne Mathilda Josephus Elissen, Hubertus Johannes Maria Vrijhoef

**Affiliations:** 1 Department of TRANZO, Faculty of Social and Behavioural Sciences, Tilburg University, PO Box 90153, 5000 LE Tilburg, The Netherlands; 2 Department of Health Services Research, CAPHRI School for Public Health and Primary Care, Maastricht University, P.O. Box 616, 6200 MD Maastricht, The Netherlands; 3 Saw Swee Hock School of Public Health, National University of Singapore and National University Health System, Singapore, Singapore

**Keywords:** Integrated care, Chronic care model, CMO model, Implementation model, Diabetes type 2, Chronic conditions

## Abstract

**Background:**

Integrated care interventions for chronic conditions can lead to improved outcomes, but it is not clear when and why this is the case. This study aims to answer the following two research questions: First, what are the context, mechanisms and outcomes of integrated care for people with type 2 diabetes? Second, what are the relationships between context, mechanisms and outcomes of integrated care for people with type 2 diabetes?

**Methods:**

A systematic literature search was conducted for the period 2003–2013 in Cochrane and PubMed. Articles were included when they focussed on integrated care and type 2 diabetes, and concerned empirical research analysing the implementation of an intervention. Data extraction was performed using a common data extraction table. The quality of the studies was assessed with the Mixed Methods Appraisal Tool. The CMO model (context + mechanism = outcome) was used to study the relationship between context factors (described by the barriers and facilitators encountered in the implementation process and categorised at the six levels of the Implementation Model), mechanisms (defined as intervention types and described by their number of Chronic Care Model (sub-)components) and outcomes (the intentional and unintentional effects triggered by mechanism and context).

**Results:**

Thirty-two studies met the inclusion criteria. Most reported barriers to the implementation process were found at the organisational context level and most facilitators at the social context level. Due to the low number of articles reporting comparable quantitative outcome measures or in-depth qualitative information, it was not possible to make statements about the relationship between context, mechanisms and outcomes.

**Conclusions:**

Efficient resource allocation should entail increased investments at the organisational context level where most barriers are expected to occur. It is likely that investments at the social context level will also help to decrease the development of barriers at the organisational context level, especially by increasing staff involvement and satisfaction. If future research is to adequately inform practice and policy regarding the impact of these efforts on health outcomes, focus on the actual relationships between context, mechanisms and outcomes should be actively incorporated into study designs.

**Electronic supplementary material:**

The online version of this article (doi:10.1186/s12913-015-1231-3) contains supplementary material, which is available to authorized users.

## Background

With health systems facing the burden of an ageing society, finding sustainable solutions for the increasing number of people with chronic conditions has become an urgent task for health practitioners and policymakers around the globe. Integrated care has been suggested as one of the solutions. The World Health Organization has described integrated care as “the management and delivery of health services such that people receive a continuum of health promotion, health protection and disease prevention services, as well as diagnosis, treatment, long-term care, rehabilitation, and palliative care services through the different levels and sites of care within the health system and according to their needs” [1].

While previous research has shown that integrated care initiatives can lead to improved outcomes for chronic conditions [2–5], this is not always the case and it is often not clear when or why certain interventions are effective [6, 7]. However, not knowing which intervention types or settings are conducive to successful implementation makes it difficult to adequately inform policymakers and practitioners regarding their choices for efficient allocation of scarce health resources.

As a solution to this, researchers have called for an increased focus on examining the implementation process of integrated care interventions and its relationship to the outcomes achieved, instead of a narrow focus on outcomes only [8–10]. It is assumed that integrated care is a form of social change, for whose evaluation the “context + mechanism = outcome model” (CMO model) has been suggested [11]. The CMO model proposes that interventions only have successful outcomes when they introduce appropriate mechanisms in the appropriate social and cultural contexts.

This study builds on a previous systematic literature review using the same search, which reported on the different types of integrated care interventions for type 2 diabetes, the outcomes achieved and the relationship between intervention type and outcomes [12]. For the purpose of this review, the concept of integrated care was linked to the Chronic Care Model (CCM), which postulates that improving integrated chronic care requires changes in four components: self-management support, delivery system design, decision support and clinical information system [13]. Intervention type was then defined as the number of CCM components included in the intervention as well as the number of sub-components as defined by a detailed operationalisation of the four CCM components (see Table 1).

**Table 1:** Operationalisation of the four CCM components adapted from Busetto et al. 2014

CCM component	CCM sub-components
Self-management support	Information provision
	Patient education – general
	Patient education – disease education
	Patient education – self-management education
	Provision of self-management tools
	Patient centeredness / involvement
	Behavioural / motivational support
	Other
Delivery system design	Team-based care provision
	Structured care
	Individualised care
	Shared care
	Medicines management
	Follow-up
	Case management
	Advanced access to health care
	Nurse-led care
	Health literacy
	Cultural sensibility
	Other
Decision support	Evidence-based guidelines
	Provider education
	Feedback
	Specialist expertise
	Non-automated performance monitoring
	Identification of barriers to care
	Non-automated reminders
	Other
Clinical information system	Patient reminder system
	Provider reminder system
	Patient registry
	Disease registry
	Automated performance monitoring
	Electronic medical record
	ICT devices
	Other

The review found that most interventions included all CCM components as well as a variety of sub-components. Moreover, most studies reported positive patient, process and health service utilisation measures. The information on costs was limited and inconsistent. Because of the low number of articles reporting effects on comparable outcome measures, no statements could be made regarding the association between intervention type and outcomes. The authors concluded that future research should focus on gaining insights into the relationships between intervention type and outcomes as well as the context factors influencing these relationships.

Based on these results, the objective of the present study is to provide a systematic overview of the contexts in which integrated care for type 2 diabetes was implemented and to provide insights into the relationship between mechanisms, contexts and outcomes. Therefore, the review aims to answer the following two research questions:

1. What are the contexts, mechanisms and outcomes of integrated care for people with type 2 diabetes?
2. What are the relationships between context, mechanisms and outcomes of integrated care for people with type 2 diabetes?

This study is part of Project INTEGRATE, which aims to investigate the leadership, management and delivery of integrated care to help European health care systems responding to the challenges of an ageing population and the increasing number of people living with chronic conditions.

## Methods

The methods of this literature review have been described in detail in a study protocol [14].

### Concepts and definitions

In line with previous research, interventions were identified as integrated care interventions when they included two or more of the four core CCM components [2, 3, 5, 15]. The four CCM components were further operationalised into four sets of sub-components (Table 1). The CMO model was used to study implementation by distinguishing between mechanism, context and outcomes [11, 16, 17]. We operationalised the concepts as follows: “Mechanism” is understood to mean the different types of integrated care, defined by the number of CCM components and sub-components they target. “Context” is defined as the setting in which the mechanisms are brought into practice. This setting can be described using the Implementation Model (IM) by Grol and Wensing, which specifies six levels of health care at which barriers and facilitators to change can occur: innovation (advantages in practice, feasibility, credibility, accessibility, attractiveness), individual professional (awareness, knowledge, attitude, motivation to change, behavioural routines), patient (knowledge, skills, attitude, compliance), social context (opinion of colleagues, culture of the network, collaboration, leadership), organisational context (organisation of care processes, staff, capacities, resources, structures) and economic and political context (financial arrangements, regulations, policies) [18]. We describe the context by detailing the barriers and facilitators to change that occur at the six levels of the IM [18]. By “outcomes” we mean the intentional and unintentional effects triggered by mechanism and context.

### Literature search and study selection

The Cochrane and PubMed databases were searched for the period 2003–2013 using the following four groups of search terms: 1. health condition; 2. intervention type; 3. CCM components; and 4. implementation. Table 2 shows the complete search terms and search string.

**Table 2:** Search terms and search string

#	Group	Search terms
#1	Diabetes	Diabetes OR DMT2
#2	Integrated Care	Integrated care OR disease management OR disease state management OR comprehensive healthcare OR complex interventions OR multifactorial lifestyle interventions OR shared care OR chronic care model OR care transition OR transitional care OR intermediate care OR case management
#3	Chronic Care Model – Self-management support	Self-management support OR self-care OR self-management OR patient-centeredness OR patient-centred care OR behavioural support OR motivational support
#4	Chronic Care Model – Delivery system design	Delivery system design OR care pathway OR critical pathway OR individualised care plan OR clinical case management services OR medicines management OR co-morbidities management OR health literacy OR cultural sensibility OR practice nurse counselling OR team-based care provision
#5	Chronic Care Model – Decision support	Decision support OR clinician reminders OR patient reminders OR provider education OR reminder systems OR specialty expertise integration OR individualised care plans
#6	Chronic Care Model – Clinical information system	Clinical information system OR clinical registry OR population information database OR shared information system OR health information systems OR health information technology OR electronic registry OR clinical reminder OR patient reminder or clinician reminder OR provider feedback OR performance monitoring OR ICT devices OR patient portal OR telemonitoring OR telehealth OR teleassistance OR telehomecare OR videoconferencing OR mobile phone OR electronic health record OR patient-held record
#7	Implementation	Implementation
#8	Complete search string	Diabetes AND ((integrated care OR (self-management support AND delivery system design) OR (self-management support AND decision support) OR (self-management support AND clinical information system) OR (delivery system design AND decision support) OR (delivery system design AND clinical information system) OR (decision support AND clinical information system)) AND implementation
		#1 AND ((#2 OR (#3 AND #4) OR ((#3 AND #5) OR ((#3 AND #6) OR (#4 AND #5) OR (#4 AND #6) OR (#5 AND #6)) AND #7

Between September 2013 and January 2014 articles were selected in three rounds based on their title, abstract and full text version. Articles were assessed independently and results were discussed in pairs (LB and KL; LB and AE) until consensus was reached.

To ensure a homogenous selection procedure, all researchers were required to use a checklist specifying in- and exclusion criteria. Articles were included when they were published between 2003 and 2013, concerned integrated care, focussed on type 2 diabetes, and concerned empirical research analysing the implementation of an intervention. They were excluded when written in a language other than English, German, Dutch, Spanish or Swedish (i.e. other than Project INTEGRATE languages), targeted populations consisting exclusively of children, adolescents, prisoners or homeless persons (i.e. populations different from Project INTEGRATE target populations), or when they did not concern empirical research. Systematic reviews and meta-analyses were excluded as well because they generally base their findings on interventions that would not necessarily all fit our definition of integrated care. For the first research question, studies had to report barriers or facilitators encountered in the implementation of the integrated care interventions. For the second research question, studies had to report barriers or facilitators as well as outcomes of the intervention.

### Data extraction and quality assessment

Data extraction was performed between September 2013 and January 2014 by LB, KL and AE using a common data extraction table specifying the following information: author, publication year, title, data collection methods, type of data, data collection setting, follow-up period, population, participants, researcher’s influence, data analysis, research questions and/or article objective, study limitations, intervention name, purpose, CCM sub-components, barriers, facilitators and outcomes [14, 19]. For each included study, the data extraction table was completed by two researchers independently and results were discussed in pairs until consensus was reached (LB and KL; LB and AE).

The Mixed Methods Appraisal Tool (MMAT), which is a unified quality assessment tool for the appraisal of qualitative, quantitative and mixed methods studies, was used to assess the methodological quality of the papers, [19, 20]. Despite its relative novelty, the MMAT has been used as a comprehensive quality assessment tool in various systematic reviews in the health sciences [21–23]. Its criteria can be fulfilled, unfulfilled or unmentioned. For each study, two researchers performed the appraisal independently and results were discussed in pairs (LB and KL; LB and AE).

### Data analysis

Barriers and facilitators were analysed based on the IM [18]. Moreover, we examined the relationships between mechanisms and context; context and outcomes; and context, mechanism and outcomes. Mechanisms were operationalised as the intervention’s number of CCM components (2, 3 or 4) and the number of CCM sub-components (1–5, 6–10, 11–15 or 16–20). Context was operationalised as the number of barriers/facilitators encountered (0–2, 3–5, 6–8 or 9–11) and the number of IM levels at which barriers/facilitators were encountered (0, 1–3 or 4–6). Outcomes included patient measure (glycaemic control, blood pressure, cholesterol), process measures (measurements of glycaemic control, blood pressure, cholesterol, foot examinations, eye examinations) and health service utilisation, which could be worsened, neutral or improved. In line with previous reviews on the effectiveness of integrated care interventions, we decided not to use pooled analyses given the large differences between the included studies regarding interventions, settings and patient populations [7, 24].

We created cross tables and performed chi-square tests to test for statistically significant relationships between the above variables. For all but three chi-square tests, the assumption that all expected cell values E must be equal to or higher than one was not fulfilled. For those three tests that did fulfil the assumptions (patient outcomes for cholesterol by number of barriers; patient outcomes for glycaemic control by number of implementation levels at which barriers were reported; and patient outcomes for cholesterol by number of implementation levels at which barriers were reported), the outcome of the chi-square test was not significant. Consequently, we opted for a more qualitative approach and examined what the studies themselves specified in terms of information on the relationships between context, mechanism and outcomes.

## Results

**Fig. 1:**
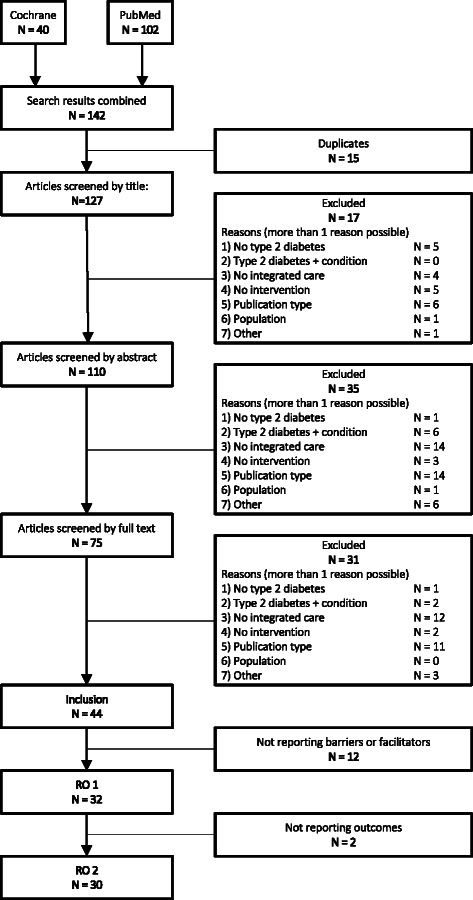
Flowchart portraying the literature review selection process. No type 2 diabetes: Article does not focus on diabetes or focusses only on type 1 diabetes. Type 2 diabetes + condition: Articles focusses on diabetes and one or more other conditions and results are not reported separately for diabetes. No integrated care: The article does not focus on integrated care as defined by targeting two or more chronic care model components. No intervention: The article does not focus on the implementation of an (integrated care) intervention. Publication type: The publication type of the article concerns a review or meta-analysis or does not concern empirical research. Population: The article targets a population consisting exclusively of children, adolescents, prisoners or homeless persons. Other: Reasons for exclusion other than the above. RO: Research objective

Figure 1 depicts a flow chart portraying the selection process.

The final selection consisted of 32 studies for research objective one (to provide a systematic overview of the contexts in which integrated care for type 2 diabetes was implemented) and 30 for research objective two (to provide insights into the relationship between mechanisms, contexts and outcomes). See Additional file 1: Table S1 for an overview of the study objective, follow-up period, setting, population, and outcomes of the included studies. Generally, studies assessed the impact of integrated care interventions on pre-specified diabetes-related outcome measures or extracted lessons from the implementation process by describing successful interventions, highlighting barriers and facilitators and reporting patient and provider experiences. Follow-up periods ranged between 1 and 96 months (median = 18). A total of 22 studies were conducted in the United States, whereas eight studies were set in the European Union, including Germany, Belgium, the Netherlands and the United Kingdom. Two studies took place elsewhere (Canada and Israel).

Additional file 2: Table S2 shows the intervention types of the included studies. Nineteen studies included all CCM components [25–43], ten studies concerned three components [44–53] and two studies targeted two components [54, 55]. One study used practice implementation of the CCM as the dependent variable without reporting specific sub-components (indicated as empty cells in Additional file 2: Table S2) [56].

### Quality assessment

Of the 32 articles, ten studies in total fulfilled all quality criteria applicable to their respective study type. Generally, it was difficult to assess whether unmentioned criteria were due to lower methodological quality or concise reporting. The three studies only fulfilling two quality criteria or less are marked with an asterisk (*) in the remainder of the article and no examples from these articles were used.

### Context

Tables 3 and 4 present the barriers and facilitators encountered in the included studies, categorised at the six levels of the IM [18].

**Table 3:** Barriers of the integrated care interventions by Implementation Model levels

Ref.	Innovation	Individual Professional	Patient	Social Context	Organisational Context	Economic & Political Context
[39]^a^	- Delayed software installation			- Competing staff priorities		
[40]^a^	- No useful outcome data				- Workflow changes	
[44]	- Wireless Internet		- Using self-management tools	- Committed staff	- Location of computer in practice	- Funding
	- Software updates			- Staff priorities		- Uncertain programme sustainability
[32]			- Unwillingness to consult experts		- Too broad referral indication	
[41]^a^						
[45]	- Unavailability of wireless Internet					- High costs
[42]				- Difficult local context		
[54]	- Lack of IT system	- Unwillingness to share care	- Unwillingness to consult experts	- Suboptimal leadership	- Information provision	- Restricting legal regulations
		- Perceived inexpertise	- Lack of motivation/compliance/knowledge	- Rivalry	- Communication	
					- Lack of (educational) structure	
[37]		- Low engagement				
		- High attrition rate				
[26]		- High attrition rate				
[33]						
[50]			- Lack of prompting	- Culture/behavioural changes	- Location of computer in practice setting	
			- Time constraints			
			- Unavailability of technology			
			- Personal factors affecting IT use			
[55]		- Resistance to messaging	- Unawareness of system features			
[25]		- Reluctance to discharge patients	- Reluctance to be discharged			
[48]		- Understanding/implementing diabetes education		- Safety issues (neighbourhoods, patients)	- Provider training	
					- Staff turnover	
					- Large caseloads	
		- Using tools			- Wide geographical area	
[27]						
[34]	- Lack of IT support		- Language and literacy problems	- Lack of leadership support	- Time constraints	
	- Manual data entry				- Limited staff capacity	
					- High staff turnover	
[28]			- Medically and socially complicated patients		- Limited staff capacity	
[29]	- Long consultations	- Reluctance to use IT		- Culturally diverse setting		
	- Translating materials					
	- Use of interpreters					
[30]	- Registry building (multiple data sources, inconsistent formatting)	- Unwillingness to share data		- Changing culture	- Changing the workflow and culture of the practice	- Funding concerns
	- Implementing/teaching change model					- Uncertain programme sustainability
[51]	- Accommodating self-management tools at home		- Inexperience with self-management tools			
[43]						
[35]						
[38]	- Lack of registry		- Lack of self-motivation		- Space limitations	
	- Difficulties in building a registry				- Time constraints	
[31]	- Lack of integrated approach to information management					
[52]		- Difficult computer use	- Difficult computer use			
[56]		- Psychosocial barriers		- Competing staff priorities		
				- Lack of openness to innovation		
[53]	- Intervention complexity	- Fear of losing patients	- Lack of patient self-motivation		- Implementing workflow changes	- Income concerns
						- Funding concerns
		- Lack of diabetes-specific expertise			- Administrative burden	- Uncertain programme sustainability
					- Isolated work	
					- Lack of staff	
[46]						
[49]	- Intervention complexity					
[36]	- Implementation of registry		- Economically complicated patients		- Implementing workflow changes	
					- Unanticipated staff changes	
[47]						

^**a**^indicates articles with lower methodological quality. Empty cells indicate that no barriers were mentioned in the category

**Table 4:** Facilitators of the integrated care interventions by Implementation Model levels

Ref.	Innovation	Individual Professional	Patient	Social Context	Organisational Context	Economic & Political Context
[39]^a^	+ Simple visual IT layout			+ Staff involvement/ cooperation		
				+ Resource-sharing		
				+ Change agent		
[40]^a^	+ Systematic identification and assignment of patients	+ Provider education		+ Leadership support	+ Multidisciplinary team	
	+ Dedicated staff time					
[44]	+ Bilinguals			+ Local champions		
	+ Translations					
[32]		+ Encouragement		+ Shared leadership		
		+ Provider education		+ Shared goals		
[41]^a^	+ Registry					
	+ Outcome data					
[45]			+ Home tutorial			
			+ Social networking			
[42]	+ Time-efficient intervention			+ Culture of change		+ Low-cost intervention
[54]						
[37]						
[26]						
[33]	+ Multifaceted intervention				+ Nurse case manager	
[50]	+ Multimedia audiovisual prompting		+ Patient instruction			
	+ Bilinguals					
[55]	+ Electronic messaging					
[25]	+ Outcome data			+ Adapting to change		
	+ Registry			+ Competition		
[48]	+ Drop-ins	+ Participatory, informal provider education			+ Job conditions	
					+ Case conferences	
					+ Shared caseload	
					+ Safety protocols	
[27]	+ Automated data extraction					
[34]	+ Registry	+ Provider education (on guidelines)		+ Changing practice culture	+ Changes to organisation’s policies and procedures	
	+ Outcome data					
	+ Multilinguals					
	+ Translations	+ Persistence		+ Staff buy-in		
	+ Pictorial focus					
[28]	+ Drop-ins	+ Ability to establish personal relationships with patients	+ Linkages between home situations and clinical care		+ Changing workflow	
	+ Varied activities					
			+ Peer support			
[47]		+ Provider reminders			+ Use of flow sheets	
[30]	+ Registry	+ Provider education			+ Changing practice workflow	
	+ Access to process outcomes					
[51]						
[43]	+ Electronic registry					+ Low-cost intervention
[35]	+ Minimal bureaucracy	+ Provider education			+ Cooperation & communication	
					+ Timely referral	
					+ Case conferences	
[38]	+ Electronic medical record		+ Patient-to-patient feedback	+ Staff buy-in		
[31]						
[52]						
[56]		+ Ability to broach delicate topics		+ Openness to innovation		
[53]		+ Provider involvement		+ Regional embeddedness		+ Availability of legal national task profiles
				+ Leadership commitment		
[46]		+ Provider education		+ Regional embeddedness	+ Guideline dissemination	
		+ Specialist support				
[49]				+ Resource-sharing		
				+ Working environment		
[36]				+ Leadership commitment		
				+ Staff involvement		
				+ Change agents		
[47]		+ Provider reminders				

^**a**^indicates articles with lower methodological quality. Empty cells indicate that no facilitators were mentioned in the category

#### Barriers

A recurring topic at the innovation level was difficulties relating to the database or electronic medical record used for the innovation, either because there was no such health IT in place, because the implementation of the health IT was problematic or because the system did not generate useful outcome data. At the individual professional level, reluctance to discharge patients or share care as well as general low provider engagement were often mentioned. Also, provider incapability or reluctance to use IT systems were often reported. Finally, lack of diabetes- or self-management-related provider expertise was also mentioned as a barrier at the individual professional level. At the patient level, several barriers related to the IT system and patients’ difficulties using the system. Other barriers related to patients’ unwillingness to be discharged, their lack of motivation or knowledge, and their medically, socially or economically complicated backgrounds.

Social context barriers included competing staff priorities, changing the culture at the workplace and suboptimal leadership. Difficult areas such as unsafe neighbourhoods or ethnically diverse settings were also among the social context barriers. Most barriers at the organisational context level related to workflow changes due to the introduction of an innovation, logistical barriers and problems relating to staff turnover or limited staff capacity. Economic- and political-context barriers mostly related to concerns regarding funding and the (financial) sustainability of an innovation, but one barrier also related to legal requirements hindering an innovation.

#### Facilitators

Facilitators at the innovation level included the use of bilinguals, translations and pictures as well as database availability and certain database features such as generation of useful outcome data. Most individual professional facilitators focussed on guidelines and disease- or self-management-related provider education. Other facilitators related to the providers’ ability to engage with patients, their motivation and the use of reminders. Patient-level facilitators included provision of patient education and peer support.

Several of the social context facilitators related to the involvement of staff in decision-making and planning, the ability to find committed staff and generate staff buy-in, good leadership and intra- as well as inter-practice resource-sharing and cooperation. The practice’s culture and openness to change were also facilitators at the social context level. Organisational context-level facilitators mostly related to multidisciplinary teamwork and workflow changes. Economic and political facilitators reported the low costs of the intervention and the availability of national task profiles.

### Context + mechanism = outcome

Even though the literature review identified a substantial evidence base on the separate components of the CMO model, only a very limited number of studies reported the actual relationships between the intervention type implemented, barriers and facilitators encountered and outcomes achieved. Thirteen studies provided information on the impact of the barriers and facilitators on intermediate output variables or other variables, but not on the outcome indicators measured in the respective studies [26, 44–47, 54]. For example, several studies mentioned how a certain facilitator improved communication or office workflow, but not how these in turn led (or did not lead) to improved patient outcome indicators as measured within the scope of the same study.

Eight studies specified the way in which barriers and facilitators encountered affected the outcomes measured. With regard to the effect of facilitators, the study by Borgermans et al. found that interdisciplinary diabetes care teams were associated with significant improvements in HbA1, LDL-cholesterol as well as increased statin and anti-platelet therapy use. According to the authors, these positive results can be explained by the quality task orientation of the team and the fact that there was shared leadership with shared group goals [38]. Gabbay et al. found that nurse case management led to reduced blood pressure mainly because the intervention was multifaceted, consisting of components such as patient education, behavioural goal setting, therapeutic adjustments and close follow-up [33]. Lemay et al. reported that a community health centre collaborative could not have led to increased patient self-management without changing the health centre philosophy towards more patient centredness and empowerment [34]. Rothe et al. attributed the success of the Saxon Diabetes Management Program in improving A1C and blood pressure to timely referral of patients to the specialised diabetes practitioners, and to the enhanced competences of general practitioners. Moreover, they claimed that the collective discussion about quality management data between health care providers from different levels of health care was pivotal for the success of the programme [35]. The low health literacy and culturally sensitive diabetes education programme studied by Swavely et al. led to significant improvements in patient knowledge, self-care behaviour, self-efficacy and A1C, and high patient, provider and staff satisfaction. According to the authors, this could not have been achieved without the creation of a non-intimidating environment [49]. Finally, Yu and Beresford found three critical success factors for their chronic illness model that led to improvements in HbA1C, blood pressure, LDL and urine albumin-to-creatinine ratio, namely leadership commitment to change, increased clinical staff involvement and residents acting as change agents [36].

Two studies reported how barriers inhibited programme success. While the web-based diabetes intervention for physicians studied by Estrada et al. was associated with an increase in A1C and LDL assessments, it did not lead to improvements in A1C control, blood pressure control or LDL control. The authors explained this lack of improvement in patient outcomes by a high attrition rate as well as low provider web engagement [37]. Sanchez found that the implementation of a diabetes self-management education programme in primary care using shared medical appointments did not lead to improvements in A1C, blood pressure and body mass index. The study found that patients without motivation for self-management tended to have a higher A1C level and were less likely to return to a follow-up shared medical appointment [38].

## Discussion

This paper has presented a literature review of the context, mechanisms and outcomes of integrated care for type 2 diabetes identified in the international literature. Most reported barriers to the implementation process were related to the organisational context level, including workflow changes due to the introduction of the integrated care initiative and logistical barriers and problems relating to staff turnover or limited staff capacity. Most facilitators to the implementation process were found at the social context level, including involvement of staff in decision-making and planning, the ability to find committed staff and generate staff buy-in, good leadership and intra- and inter-practice resource-sharing and cooperation. It is difficult to say whether these findings are in line with previous reviews of integrated care for type 2 diabetes as these have typically focussed on the effect of the intervention on outcomes, sometimes assessing the relative effectiveness of different intervention components [3, 24, 57–59]. None of these reviews, however, focussed on barriers and facilitators to the implementation process and/or their potential mediating effect on the relationship between interventions and outcomes. A previous review by Renders identified barriers to change in diabetes care, which included a lack of guideline acceptance, a lack of diabetes knowledge, poor staff member cooperation, poor quality care documentation, guideline complexity and a lack of information needed to incorporate these guidelines into practice, non-attendance and poor patient compliance. However, these were barriers identified prior to the implementation of the intervention instead of barriers encountered during the implementation process, as was the focus of the present study.

Our findings regarding the occurrence of most barriers at the organisational context level suggest that if targeted policy programmes and quality improvement strategies are to yield the most significant impact, efficient allocation of health resources should entail more resources allocated to the organisational context to provide additional support in those areas where most obstacles are expected to occur. At the same time, this should not occur at the expense of investments at the social context level because although most facilitators to the implementation process were encountered at this level, investments for sufficient resources are needed to benefit optimally from those factors that help the implementation process to develop relatively smoothly. It is also likely that investments in the social context level to increase staff involvement and satisfaction will decrease the development of barriers at the organisational context level, such as staff turnover and limited staff capacity.

Our ability to make statements about the relationships between context, mechanisms and outcomes was severely impeded by the low number of articles reporting comparable quantitative outcome data as well as the small amount of articles reporting in-depth qualitative information on the relationships between context, mechanisms and outcomes. Only eight studies qualitatively described the interplay between context, mechanisms and outcomes, but due to the lack of previous reviews focussing on barriers and facilitators to the implementation process, we cannot say how these findings relate to previous research on integrated care for type 2 diabetes.

There are several limitations associated with this study that should be taken into consideration. First, there are various definitions and conceptualisations of integrated care and the decision to link integrated care to the CCM is therefore not undisputed. However, for the specific purpose of this review, an operational definition was needed that could be applied structurally and uniformly to the identification of integrated care interventions from the literature. As mentioned above, the CCM has been used to this end repeatedly in the literature [2–5, 60]. The question was also posed to an expert committee from Project INTEGRATE, but its members could not provide a feasible alternative operational definition and eventually consensus was reached for our approach.

The second limitation relates to the quality assessment instrument. The MMAT is a comprehensive quality assessment tool that allows for the simultaneous assessment of qualitative, quantitative and mixed methods studies [19]. However, based on the MMAT it was often not possible to determine whether unfulfilled or unmentioned criteria were a sign of substandard methodology or concise reporting. Fortunately, the information reported by the three studies with only two fulfilled criteria or less did not differ from the information reported by the other articles. Therefore, it is unlikely that the inclusion of these studies biased the findings of this paper.

The third limitation concerns the data extraction for the barriers and facilitators. The authors chose to only include information on those barriers and facilitators that were explicitly identified as such by the authors of the included studies. Of course, different authors may have been more or less exhaustive in explaining the reasons for the success or failure of their interventions and our findings may be biased accordingly. Nevertheless, most authors did encounter barriers and facilitators and chose to report those most pertinent to their findings. Therefore, the choice was made to consider the studies’ authors as experts of their own study and to follow their observations as the most reliable source of information on barriers and facilitators.

The strength of this article lies in its embeddedness in three robust and widely used theoretical models. The CMO made it possible to look at the context, mechanisms and outcomes of integrated care as separate elements as well as a complex, collective web of interrelationships between the three separate elements. The CCM helped to identify and categorise different types of integrated care interventions despite the lack of a common conceptual definition of integrated care and the use of different operational definitions of integrated care in the included studies. With the IM a diverse array of barriers and facilitators could be categorised and analysed. However, the very low number of articles reporting comparable outcome measures made it difficult to statistically analyse the relationship between context, mechanism and outcomes, and while the qualitative insights provided in the studies are informative, they remain extremely limited. This means that while we do know in which areas most barriers and facilitators can be expected to occur, we do not know their expected impact on health outcomes. Nor do we know whether certain intervention types make it more likely that certain barriers or facilitators will (or will not) be encountered or what their combined effect on outcomes would be. This means that while there is ample separate information on the context, mechanisms and outcomes of integrated care for type 2 diabetes, there is neither enough of the same quantitative information to statistically analyse the relationships between these parts, nor is there enough qualitative information to provide meaningful insights into how the separate parts are linked. Consequently, more CMO-informed focus on the actual relationships between context, mechanisms and outcomes must be actively incorporated into study designs if future research is to adequately inform practitioners and policymakers regarding their choices on efficient resource allocation for integrated care interventions.

## Conclusions

This systematic review of the context, mechanisms and outcomes of integrated care interventions for type 2 diabetes found most reported barriers to the implementation process to be related to the organisational context and most facilitators to be related to the social context level. Based on the insights of this review it is suggested that efficient allocation of health resources should entail more resources allocated to the organisational context to provide additional support in those areas where most obstacles are expected to occur. Moreover, it is likely that investments at the social context level, especially to increase staff involvement and satisfaction, will also help to decrease the likelihood of barriers occurring at the organisational context level. Due to the limited number of studies reporting comparable outcomes measures as well as the low number of articles reporting relevant qualitative information, it was not possible to make statements about how the context and mechanisms of the integrated care interventions for type 2 diabetes influenced outcomes achieved. As retrospectively linking the separate elements of the CMO model is therefore not possible, future research should be conducted with the CMO model incorporated into study designs so as to gain insights into the relationships between the context, mechanisms and outcomes of integrated care.

## Additional files

Additional file 1: **Study objective, follow-up period, setting, population, and outcomes of the included studies.** (DOCX 68 kb)

Additional file 2: **Detailed Chronic Care Model Classification.** (DOC 199 kb)

## Abbreviations

CCM: chronic care model
CMO model: Context + mechanism = outcome model
IM: implementation model
MMAT: mixed methods appraisal tool

## References

[1] World Health Organization. Roadmap Strengthening people-centred health systems in the WHO European Region. A framework for Action towards Coordinated/Integrated Health Services Delivery (CIHSD). Copenhagen, Denmark: WHO Regional Office for Europe; 2013.

[2] Drewes HW, Steuten LM, Lemmens LC, Baan CA, Boshuizen HC, Elissen AM (2012). The effectiveness of chronic care management for heart failure: meta-regression analyses to explain the heterogeneity in outcomes. Health Serv Res.

[3] Elissen AMJ, Steuten LMG, Lemmens LC, Drewes HW, Lemmens KMM, Meeuwissen JAC (2012). Meta-analysis of the effectiveness of chronic care management for diabetes: investigating heterogeneity in outcomes. J Eval Clin Pract.

[4] Lemmens KM, Lemmens LC, Boom JH, Drewes HW, Meeuwissen JA, Steuten LM (2013). Chronic care management for patients with COPD: a critical review of available evidence. J Eval Clin Pract.

[5] Meeuwissen JAC, Lemmens LC, Drewes HW, Lemmens KMM, Steuten LMG, Elissen AMJ, Vrijhoef HJM, Baan CA. Meta-analysis and meta-regression analyses explaining heterogeneity in outcomes of chronic care management for depression: implications for person-centered mental healthcare. The International Journal of Person Centered Medicine. 2012;2(4):716–58.

[6] Norris SL, Nichols PJ, Caspersen CJ, Glasgow RE, Engelgau MM, Jack L (2013). The effectiveness of disease and case management for people with diabetes: A systematic review. Am J Prev Med.

[7] Renders CM, Valk GD, Griffin SJ, Wagner E, Eijk JTv, Assendelft WJ. Interventions to improve the management of diabetes mellitus in primary care, outpatient and community settings (Review). The Cochrane Database of Systematic Reviews. 2001;1:CD001481. 10.1002/14651858.CD001481 PMC7045779 11279717

[8] Berwick DM (2008). The science of improvement. J Am Med Assoc.

[9] Ling T, Brereton L, Conklin A, Newbould J, Roland M (2012). Barriers and facilitators to integrating care: experiences from the English Integrated Care Pilots. International Journal of Integrated Care.

[10] Mc Hugh S, O’Mullane M, Perry IJ, Bradley C (2013). Barriers to, and facilitators in, introducing integrated diabetes care in Ireland: a qualitative study of views in general practice. BMJ Open.

[11] Pawson R, Tilley N (1997). Realistic evaluation.

[12] Busetto L, Luijkx KG, Elissen AMJ, Vrijhoef HJM (2015). Intervention types and outcomes of integrated care for diabetes mellitus type 2: a systematic review. Journal of Evaluation in Clinical Practice.

[13] Improving Chronic Illness Care. The Chronic Care Model. http://improvingchroniccare.org/index.php?p=The_Chronic_Care_Model&s=2. Accessed 8 Nov 2013.

[14] Busetto L, Luijkx KG, Vrijhoef HJM (2014). Implementation of integrated care for type 2 diabetes: a protocol for mixed methods research. International Journal of Integrated Care.

[15] Wagner EH (1998). Chronic disease management: what will it take to improve care for chronic illness?. Eff Clin Pract.

[16] Greenhalgh T, Humphrey C, Hughes J, MacFarlane F, Butler C, Pawson R (2009). How Do You modernize a health service? A realist evaluation of whole-scale transformation in London. Milbank Q.

[17] Pawson R, Greenhalgh T, Harvey G, Walshe K (2005). Realist review - a new method of systematic review designed for complex policy interventions. J Health Serv Res Policy.

[18] Grol R, Wensing M (2004). What drives change? Barriers to and incentives for achieving evidence-based practice. Med J Aust.

[19] Pace R, Pluye P, Bartlett G, Macaulay AC, Salsberg J, Jagosh J (2012). Testing the reliability and efficiency of the pilot Mixed Methods Appraisal Tool (MMAT) for systematic mixed studies review. Int J Nurs Stud.

[20] Pluye P, Gagnon M-P, Griffiths F, Johnson-Lafleur J (2009). A scoring system for appraising mixed methods research, and concomitantly appraising qualitative, quantitative and mixed methods primary studies in Mixed Studies Reviews. Int J Nurs Stud.

[21] Bluebond-Langner M, Beecham E, Candy B, Langner R, Jones L (2013). Preferred place of death for children and young people with life-limiting and life-threatening conditions: A systematic review of the literature and recommendations for future inquiry and policy. Palliat Med.

[22] Langston B, Armes J, Levy A, Tidey E, Ream E (2013). The prevalence and severity of fatigue in men with prostate cancer: a systematic review of the literature. Support Care Cancer.

[23] Pedersen VH, Armes J, Ream E (2012). Perceptions of prostate cancer in black African and black Caribbean men: a systematic review of the literature. Psychooncology.

[24] Fokkens AS, Wiegersma PA, Reijneveld SA (2011). Organization of diabetes primary care: a review of interventions that delegate general practitioner tasks to a nurse. J Eval Clin Pract.

[25] Heymann AD, Chodick G, Halkin H, Karasik A, Shalev V, Shemer J (2006). The implementation of managed care for diabetes using medical informatics in a large Preferred Provider Organization. Diabetes Res Clin Pract.

[26] Flamm M, Panisch S, Winkler H, Johansson T, Weitgasser R, Sonnichsen AC (2012). Effectiveness of the Austrian disease management programme “Therapie Aktiv” for type 2 diabetes regarding the improvement of metabolic control, risk profile and guideline adherence: 2 years of follow up. Wien Klin Wochenschr.

[27] Hunt JS, Siemienczuk J, Gillanders W, LeBlanc BH, Rozenfeld Y, Bonin K (2009). The impact of a physician-directed health information technology system on diabetes outcomes in primary care: a pre- and post-implementation study. Inform Prim Care.

[28] Liebman J, Heffernan D, Sarvela P (2007). Establishing diabetes self-management in a community health center serving low-income Latinos. Diabetes Educ.

[29] Nasmith L, Cote B, Cox J, Inkell D, Rubenstein H, Jimenez V (2004). The challenge of promoting integration: conceptualization, implementation, and assessment of a pilot care delivery model for patients with type 2 diabetes. Fam Med.

[30] Nuovo J, Balsbaugh T, Barton S, Davidson E, Fox-Garcia J, Gandolfo A (2004). Development of a diabetes care management curriculum in a family practice residency program. Dis Manag.

[31] Simmons D, Yu D, Wenzel H (2013). Changes in hospital admissions and inpatient tariff associated with a Diabetes Integrated Care Initiative: Preliminary findings. J Diabetes.

[32] Borgermans L, Goderis G, Van Den Broeke C, Verbeke G, Carbonez A, Ivanova A (2009). Interdisciplinary diabetes care teams operating on the interface between primary and specialty care are associated with improved outcomes of care: findings from the Leuven Diabetes Project. BMC Health Serv Res.

[33] Gabbay RA, Lendel I, Saleem TM, Shaeffer G, Adelman AM, Mauger DT (2006). Nurse case management improves blood pressure, emotional distress and diabetes complication screening. Diabetes Res Clin Pract.

[34] Lemay CA, Beagan BM, Ferguson WJ, Hargraves JL (2010). Lessons learned from a collaborative to improve care for patients with diabetes in 17 community health centers, Massachusetts, 2006. Prev Chronic Dis.

[35] Rothe U, Muller G, Schwarz PE, Seifert M, Kunath H, Koch R (2008). Evaluation of a diabetes management system based on practice guidelines, integrated care, and continuous quality management in a Federal State of Germany: a population-based approach to health care research. Diabetes Care.

[36] Yu GC, Beresford R (2010). Implementation of a chronic illness model for diabetes care in a family medicine residency program. J Gen Intern Med.

[37] Estrada CA, Safford MM, Salanitro AH, Houston TK, Curry W, Williams JH, Ovalle F, Kim Y, Foster P, Allison JJ. A web-based diabetes intervention for physician: a cluster-randomized effectiveness trial. International journal for quality in health care : journal of the International Society for Quality in Health Care/ISQua2011. p. 682–89. 10.1093/intqhc/mzr053 PMC3247785 21831967

[38] Sanchez I (2011). Implementation of a diabetes self-management education program in primary care for adults using shared medical appointments. Diabetes Educ.

[39] Alexander S, Frith KH, O’Keefe L, Hennigan MA (2011). Implementation of customized health information technology in diabetes self management programs. Clinical Nurse Specialist.

[40] Antoline C, Kramer A, Roth M (2011). Implementation and methodology of a multidisciplinary disease-state-management program for comprehensive diabetes care. The Permanente Journal.

[41] Cannon HE (2005). Best practices in innovative type 2 diabetes program management: a case study. J Manag Care Pharm.

[42] Caruso LB, Clough-Gorr KM, Silliman RA (2007). Improving quality of care for urban older people with diabetes mellitus and cardiovascular disease. J Am Geriatr Soc.

[43] Peterson KA, Radosevich DM, O’Connor PJ, Nyman JA, Prineas RJ, Smith SA (2008). Improving diabetes care in practice: Findings from the TRANSLATE trial. Diabetes Care.

[44] Bolin JN, Ory MG, Wilson AD, Salge L (2013). Diabetes education kiosks in a latino community. Diabetes Educ.

[45] Carter EL, Nunlee-Bland G, Callender C. A patient-centric, provider-assisted diabetes telehealth self-management intervention for urban minorities. Perspectives in health information management/AHIMA, American Health Information Management Association2011. p. 1b. PMC3035826 21307985

[46] Sunaert P, Bastiaens H, Nobels F, Feyen L, Verbeke G, Vermeire E (2010). Effectiveness of the introduction of a Chronic Care Model-based program for type 2 diabetes in Belgium. BMC Health Serv Res.

[47] Zgibor JC, Rao H, Wesche-Thobaben J, Gallagher N, McWilliams J, Korytkowski MT (2004). Improving the quality of diabetes care in primary care practice. J Healthc Qual.

[48] Hill-Briggs F, Batts-Turner M, Gary TL, Brancati FL, Hill M, Levine DM (2007). Training community health workers as diabetes educators for urban African Americans: value added using participatory methods. Prog Community Health Partnersh.

[49] Swavely D, Vorderstrasse A, Maldonado E, Eid S, Etchason J (2013). Implementation and evaluation of a low health literacy and culturally sensitive diabetes education program. J Healthc Qual.

[50] Gerber BS, Brodsky IG, Lawless KA, Smolin LI, Arozullah AM, Smith EV (2005). Implementation and evaluation of a low-literacy diabetes education computer multimedia application. Diabetes Care.

[51] Palmas W, Shea S, Starren J, Teresi JA, Ganz ML, Burton TM (2010). Medicare payments, healthcare service use, and telemedicine implementation costs in a randomized trial comparing telemedicine case management with usual care in medically underserved participants with diabetes mellitus (IDEATel). J Am Med Inform Assoc.

[52] Simon AC, Holleman F, Gude WT, Hoekstra JB, Peute LW, Jaspers MW (2013). Safety and usability evaluation of a web-based insulin self-titration system for patients with type 2 diabetes mellitus. Artif Intell Med.

[53] Sunaert P, Bastiaens H, Feyen L, Snauwaert B, Nobels F, Wens J (2009). Implementation of a program for type 2 diabetes based on the Chronic Care Model in a hospital-centered health care system: “the Belgian experience”. BMC Health Serv Res.

[54] Eijkelberg IM, Spreeuwenberg C, Wolffenbuttel BH, van Wilderen LJ, Mur-Veeman IM (2003). Nurse-led shared care diabetes projects: lessons from the nurses’ viewpoint. Health Policy.

[55] Hess R, Bryce CL, Paone S, Fischer G, McTigue KM, Olshansky E (2007). Exploring challenges and potentials of personal health records in diabetes self-management: implementation and initial assessment. Telemedicine journal and e-health.

[56] Strickland PA, Hudson SV, Piasecki A, Hahn K, Cohen D, Orzano AJ (2010). Features of the Chronic Care Model (CCM) associated with behavioral counseling and diabetes care in community primary care. The Journal of the American Board of Family Medicine.

[57] Mattke S, Seid M, Mat S (2007). Evidence for the effect of disease management: Is $1 billion a year a good investment?. Am J Manag Care.

[58] Pimouguet C, Goff ML, Thiébaut R, Dartigues JF, Helmer C (2011). Effectiveness of disease-management programs for improving diabetes care: a meta-analysis. Can Med Assoc J.

[59] Shojania KG, Ranji SR, McDonald KM, Grimshaw JM, Sundaram V, Rushakoff RJ (2006). Effects of quality improvement strategies for type 2 diabetes on glycemic control: A meta-regression analysis. The Journal of the American Medical Association.

[60] Elissen AMJ (2013). Going beyond the ‘grand mean’: Advancing disease management science and evidence. Dissertation.

