# Supplementary material for: Context, mechanisms and outcomes of integrated care for diabetes mellitus type 2: a systematic review
Source: BMC Health Serv Res. 2016 Jan 15;16:18. doi: 10.1186/s12913-015-1231-3 (PMC4715325; doi:10.1186/s12913-015-1231-3)
Supplement: Supplementary file 1 — Study objective, follow-up period, setting, population, and outcomes of the included studies. (DOCX 68 kb) [file 12913_2015_1231_MOESM1_ESM.docx]

**Additional file 1: Table S1: Study objective, follow-up period, setting, population, and outcomes of the included studies**

| **Ref**. | **Study objective** | **Time** | **Setting** | **Population** | **Patient outcomes** | **Process outcomes** | **Health services utilisation** | **Costs** |
| --- | --- | --- | --- | --- | --- | --- | --- | --- |
| [39]* | Not specified | 12 | - South-Eastern USA - small practice | - Diabetes patients - ≥19 years |  | **+** 100% patient record inclusion in database  **+** Strong post-test agreement with usefulness of software programs by the staff |  | **+** Project expenses totalled slightly more than half of budgeted amounts   - Most costs were incurred for staff salaries for training sessions |
| [40]* | Not specified | 12 | - Ohio, USA - Medical centres | - Diabetes patients - 18-80 years | **+** Improvement in glycaemic control, blood pressure control, lipid level control | **+** Improved screening frequency for glycaemic control | **+** Decreases in several diabetes-related clinical hospital admission parameters |  |
| [44] | To examine the implementation, use, and sustainability of a computerised touch-screen diabetes education kiosk | 11 | - Texas, USA - Pharmacies, community centre, clinics | - Diabetes patients | **+** High self-reported belief in usefulness of system and intent to change lifestyle |  |  |  |
| [32] | To create the basis for the development of a national diabetes care programme | 18 | - Leuven, Belgium - Primary care | - All primary care physicians - All DMT2 patients | **+** Significant improvement in HbA1c, LDL-cholesterol  **+** Significantly increased statin use  **+** Significantly increase anti-platelet therapy use |  | **+** Significantly higher use of interdisciplinary diabetes care teams |  |
| [41]* | To illustrate a successful DMT2 management programme | 48 | - Salt Lake City, USA - Hospitals and medical facilities | - DMT2 patients | **+** Improvement in A1c levels and LDL-C levels | **+** Improved identification of type 2 diabetes patients |  |  |
| [45] | To describe the design, implementation and outcomes of a pilot self-management intervention | 9 | - Washington, DC, USA - Primary care | - DMT2 patients - ≥18 years - African American | **+** Significant association between programme participation and achieving A1c targets  **+** Significant association between programme participation and achieving BMI targets  **+** Increased diabetes knowledge  **+** Improved perceived mental and physical health status   - No association between programme participation and achieving blood pressure targets | **+** Improved adherence to sound diabetes management practices |  | - Costs of providing each patient with a laptop and Internet access |
| [42] | Not specified | 39 | - Boston, USA - Geriatric ambulatory practice | - Patients with cardio-vascular disease, diabetes or both | **+** Association between intervention and HbA1c decrease | **+** Significantly improved frequency of HbA1c measurement  **+** Improved frequency of foot examinations  **+** Improved frequency of lipid testing |  |  |
| [54] | To explore nurses’ experiences with shared care | 36 | - Maastricht and North Limburg, The Netherlands - GP practices | - Nurse practitioners, GPs and endocrinologists | **+** Improved patient satisfaction and health status | **+** More efficient care delivery |  |  |
| [37] | To test a multi-component intervention designed to improve diabetes care | 24 | - Rural South-Eastern, USA - Primary care, internists | - Diabetes patients | - No improvements in A1c control, blood pressure control and LDL control | **+** Improvement in frequency of A1c assessment  **+** Improvement in frequency of LDL assessments   - No Improvement in frequency of blood pressure assessment |  |  |
| [26] | To assess the prolonged impact of the DMP regarding HbA1c reduction and process quality | 24 | - Salzburg, Austria - GPs' and internists' surgeries | - General physicians and internists - DMT2 patients - ≥18 years | **+** Significant improvements in HbA1c, triglycerides, cholesterol, HDL-cholesterol, LDL-cholesterol, body mass index, blood pressure | **+** Significant improvement in eye examination rate  **+** Significant improvement in foot examination rate  **-** reduced frequency of regular HbA1c checks | **+** Significantly higher participation in patient education |  |
| [33] | To measure the impact of a patient-oriented structured approach to care coordination and patient education & counseling | 12 | - Pennsylvania, USA - Primary care | - Diabetes patients - ≥18 years, | **+** Statistically significant improvement in blood pressure  **+** Significant improvement in diabetes-related distress   - No significant change in A1C, LDL and weight | **+** Significant improvement in the percentage of patients having completed ophthalmological exams, foot exams, micro-albuminuria screening, pneumonia vaccination, dietician visit, certified diabetes nurse educator visit, smoking cessation counselling |  |  |
| [50] | To evaluate the impact of computer-based education and to evaluate the barriers and facilitators to the implementation | 17 | - USA - Urban outpatient clinics | - Diabetes patients - ≥18 years | **+** Improved perceived susceptibility to complications  **+** Self-reported improvements to lifestyle changes (diet, exercise, smoking cessation, cutting nails and home glucose monitoring)   - No significant change in A1C, body mass index or blood pressure - No significant change in self-efficacy, knowledge and medical care |  |  |  |
| [55] | To describe the integration of multiple electronic systems into one patient portal, to describe the integration of the system into the outpatient office workflow, and to discuss patients’ initial reaction | 28 | - Pittsburgh. USA - Primary care | - Diabetes patients | **+** Positive patient evaluation (empowerment, better understanding of lifestyle choices)  **-** Negative patient evaluation: system inefficiencies (missing lab results, inaccurate information, slow provider responses) |  | - No change in number of patients seen - No change in number of phone calls received |  |
| [25] | To describe the reorganisation of diabetes care using disease management principles on a countrywide basis | 48 | - Israel - Private physicians’ clinics | - Diabetes patients | **+** Improvement in HbA1C and LDL | **+** Improvements in 24 h urinary collection for micro-albumin or urinary creatinine/ albumin ratio, prescription of angiotensin converting enzyme inhibitor or angiotensin receptor blockers  **+** Uptake in LDL and HbA1c testing |  |  |
| [48] | To describe the CHW recruitment, training, and evaluation procedures utilised in Project Sugar 2 | 36 | - Baltimore, USA - Primary care | - Community health workers - Patients: African American adults with DMT2 | **+** High patient satisfaction with community health workers and with intervention |  |  |  |
| [27] | To determine the effectiveness of automating multiple physician-directed diabetes interventions | 24 | - Oregon, USA - Community-based primary care | - Diabetes patients - ≥18 years | **+** Significant improvement in LDL goal attainment and mean LDL  **+** Significant improvement in blood pressure goal attainment and mean blood pressure  **+** Significantly higher number of patients below HbA1c target   - No improvement in mean HbA1c - High patient satisfaction, but no improvement to patients satisfaction | **+** Significant improvement in frequency of LDL tests, HbA1c tests, prescription for lipid lowering, ACEI/ARB, oral hypoglycaemic therapies, anti-platelet therapies, retinal and foot examinations |  | **+** Combined positive effect on annual revenue from changes in office visit frequency and coding complexity: $546 864 (year 1) and $427 776 (year 2) |
| [34] | To identify specific strategies for improvement that contributed to the intervention's success | 13 | - Massachusetts, USA - Community health centres | - Diabetes patients - Team leaders from all community health centres, provider champions, team members | **+** Improved likelihood of self-management by patients | **+** Improved self-management support by providers  **+** Improved follow-up |  |  |
| [28] | To report on the experience of a community health centre in implementing the CCM | 84 | - Holyoke, Massachusetts, USA - Community-based primary care | - Diabetes patients | **+** Improvement in average HbA1c | - Participation in self-management activities |  | **+** Relatively low cost |
| [29] | To describe the development and implementation process of the new delivery system and to describe the preliminary findings | 12 | - Côtes-de-Neiges, Quebec, Canada - Community-based family physician practices | - Diabetes patients - Family physicians, project team, project managers, members of the steering committee and advisory board | **+** Patient and provider satisfaction  **+** (Perceived) improved diabetes & lifestyle knowledge |  | **-** Low use of educational sessions & CD-ROMs by physicians  **-** Low impact of outreach activities | - Cost of hiring a sufficient number of nurses and other diabetes educators to serve a large multi-ethnic population |
| [30] | To describe the implementation of a diabetes team, to discuss the methods used to overcome the described barriers, and to provide a qualitative assessment of learners’ evaluation | n.s. | - USA - Family practice clinic | n.s. | **+** Residents felt more successful in their self-management support  **+** Residents felt more prepared to diagnose and treat diabetes |  |  |  |
| [51] | To assess the effect of the telemedicine case management on health resources utilisation | 96 | - New York City, USA - Primary care | - Diabetes patients - ≥55 years - Medicare beneficiaries - Residence in federally designated medically underserved area |  |  |  | - No significant difference in payments for all services combined between the telemedicine and usual care groups - Intervention costs estimated US $622 per participant/ month of intervention delivered - No cost savings through lower expenditures in inpatient care or substitution of electronically delivered case management services for in-person services |
| [43] | To test whether implementation of an organizational intervention could improve diabetes care processes and clinical outcomes | 12 | - Minnesota, USA - Primary care | - DMT2 patients - 18–89 years | **+** Significant improvement in blood pressure, A1C and LDL | **+** Significant improvement in frequency of blood pressure monitoring, renal, LDL & A1C testing, eye & foot examinations |  |  |
| [35] | To elaborate on the experiences gained during the implementation and evaluation of the integrated SDMP | 24 + 12 | - Saxony, Germany - GP practices, diabetes specialised practices | - Diabetes patients | **+** Improvements in A1C and blood pressure  **+** Association between timely referral and optimal A1C and blood pressure | **+** Improvement in frequency of blood pressure measurement and A1C measurement rates   - No improvement in frequency of patient consultations |  |  |
| [38] | To implement DSME and SMA to provide evidence based interventions to improve process and measure outcomes | 3 | - South Texas, USA - Internal medicine clinic | - Adults with DMT2 | - No difference in A1C, cholesterol, triglycerides, LDL |  |  |  |
| [31] | To compare changes in hospitalisation and total tariff paid | 12 | - East Cambridgeshire and Fenland, UK - GP practices and diabetes specialist service | - GP practices |  |  | **-** Increase in monthly diabetes-related hospital admission | **+** Decrease in inpatient monthly tariff change increase |
| [52] | To establish whether DMT2 patients can safely use PANDIT at home | 1 | - Amsterdam, The Netherlands | - Adult DMT2 patients |  |  |  |  |
| [56] | To examine whether offices incorporating more CCM features deliver better diabetes care and more counselling for diet or weight loss and physical activity | 24 | - New Jersey, USA - Community-based primary care | - Diabetes and overweight patients - ≥ 50 years |  | **+** Correlation between Decision Support and Behavioural Change Support  **+** Increased CCM implementation is associated with increased assessment of and treatment for HbA1c, lipids and blood pressure |  |  |
| [53] | To explore how CCM components can be implemented in a primary health care system with limited structure | 48 | - Region of Aalst, Flanders, Belgium - Primary care | - All DMT2 patients - All diabetes care providers |  |  |  |  |
| [46] | To explore ways to adapt the primary health care system to a more chronic care-oriented system | 48 | - Belgium - Primary care | - All DMT2 patients | **+** Significant improvement in HbA1c  **+** Significant improvement in cholesterol | **+** Significant increase in the percentage of patients receiving HbA1c assessment, urine test, statin therapy prescription and influenza vaccination   - No difference in total cholesterol, micro-albuminuria test and ophthalmologist visit |  |  |
| [49] | To evaluate the effectiveness of the LHL diabetes education programme | 12 | - Pennsylvania, USA - Primary care | - DMT2 patients - ≥18 years | **+** Significant improvement in patient knowledge, self-care behaviour, self-efficacy, A1C  **+** High patient, provider and education staff satisfaction |  |  | **+** Cost-effective approach to programme implementation through resource-sharing between network practices |
| [36] | To describe the development and implementation of our diabetes management programme, present clinical and educational outcomes, and provide a qualitative assessment of our learning process | 12 | - California, USA - Outpatient clinic | - Adult DMT2 patients | **+** Improvement in HbA1c, BP, LDL, urine albumin-to-creatinine ratio | **+** Improvements in provider performance report reviews, self-management support strategies & goal-setting, dilated eye & foot exams, and medication use |  |  |
| [47] | To investigate the feasibility of translating specialist diabetes care processes into primary care practice | 12 | - Pittsburgh, USA - Primary care | - Diabetes patients | **+** Significant improvement in HbA1c | **+** Improvements in frequency of HbA1c testing, annual screening for urine albumin   - No difference in frequency of performance of creatinine or foot examinations   - Significantly lower likelihood of having annual lipid profile |  |  |

Notes*:* Empty cells indicate no information reported for this category; time: follow-up time; + indicates positive outcomes or improvements; - indicates negative outcomes or deterioration; ● indicates neutral outcomes or no improvement; * indicates articles with lower methodological quality. (12)
