# Supplementary material for: Context, mechanisms and outcomes of integrated care for diabetes mellitus type 2: a systematic review
Source: BMC Health Serv Res. 2016 Jan 15;16:18. doi: 10.1186/s12913-015-1231-3 (PMC4715325; doi:10.1186/s12913-015-1231-3)
Supplement: Supplementary file 2 — Detailed Chronic Care Model Classification. (DOC 199 kb) [file 12913_2015_1231_MOESM2_ESM.doc]

**Additional file 2: Table S2: Detailed Chronic Care Model Classification**

| **Ref**. | **Self-Management** | | | | | | | | **Delivery System Design** | | | | | | | | | | | | **Decision Support** | | | | | | | | **Clinical Information System** | | | | | | | |
| --- | --- | --- | --- | --- | --- | --- | --- | --- | --- | --- | --- | --- | --- | --- | --- | --- | --- | --- | --- | --- | --- | --- | --- | --- | --- | --- | --- | --- | --- | --- | --- | --- | --- | --- | --- | --- |
|  | Information provision | Patient education – General | Patient education – Disease education | Patient education – SMGMT education | Provision of SMGMT tools | Patient centredness / involvement | Behavioural / Motivational support | Other | Team-based care provision | Structured care | Individualised care | Shared care | Medicines management | Follow-up | Case management | Advanced access to health care | Nurse-led care | Health literacy | Cultural sensibility | Other | Evidence-based guidelines | Provider education | Feedback | Specialists | Non-automated performance monitoring | Identification of barriers to care | Non-automated reminders | Other | Patient reminder system | Provider reminder system | Patient registry | Disease registry | Automated performance monitoring | EMR | ICT devices | Other |
| [39]* |  | **x** |  |  |  |  |  |  |  |  |  |  |  | **x** |  |  | **x** |  |  |  | **x** | **x** |  |  |  |  |  |  |  |  | **x** |  |  |  |  |  |
| [40]* |  |  | **x** | **x** |  | **x** | **x** |  | **x** | **x** | **x** | **x** | **x** | **x** |  |  | **x** |  |  | **x** | **x** | **x** | **x** |  |  |  |  |  | **x** |  |  |  | **x** | **x** |  |  |
| [44] | **x** |  | **x** | **x** | **x** |  |  |  |  |  |  |  | **x** |  |  |  |  | **x** | **x** |  |  |  |  |  |  |  |  |  |  |  |  |  |  |  | **x** |  |
| [32] |  |  | **x** | **x** | **x** |  |  |  | **x** | **x** |  | **x** | **x** |  |  |  |  |  |  | **x** | **x** | **x** | **x** |  |  |  |  | **x** |  | **x** |  |  |  |  |  |  |
| [41]* | **x** |  | **x** |  | **x** |  | **x** | **x** | **x** | **x** |  |  | **x** |  | **x** |  |  |  |  | **x** | **x** |  | **x** |  |  |  |  |  | **x** |  | **x** |  | **x** |  | **x** |  |
| [45] |  | **x** | **x** | **x** | **x** | **x** | **x** | **x** | **x** |  | **x** |  |  |  |  | **x** |  |  | **x** |  |  |  |  |  |  |  |  |  |  |  |  |  |  | **x** | **x** |  |
| [42] | **x** |  | **x** |  | **x** |  |  |  | **x** | **x** |  |  |  |  |  |  |  |  | **x** |  | **x** | **x** | **x** |  |  |  | **x** |  |  |  |  |  | **x** | **x** |  |  |
| [54] |  |  |  |  |  |  |  |  | **x** | **x** |  |  |  |  |  |  |  |  |  | **x** |  |  |  | **x** |  |  |  |  |  |  |  |  |  |  |  |  |
| [37] |  |  |  |  |  |  |  | **x** |  | **x** |  |  |  |  |  |  |  |  |  |  | **x** |  |  |  |  |  |  | **x** |  | **x** |  |  | **x** |  |  |  |
| [26] |  |  | **x** |  |  | **x** |  |  | **x** | **x** | **x** |  |  | **x** |  |  |  |  |  |  | **x** | **x** |  |  |  |  |  |  |  |  |  | **x** |  |  |  |  |
| [33] |  |  | **x** | **x** |  | **x** | **x** |  | **x** | **x** | **x** | **x** |  | **x** | **x** |  |  |  |  |  | **x** | **x** |  | **x** |  |  |  |  |  |  |  | **x** | **x** |  |  |  |
| [50] |  | **x** | **x** | **x** |  |  |  | **x** |  |  | **x** |  |  |  |  |  |  | **x** | **x** |  |  |  |  |  |  |  |  |  |  |  |  |  |  |  | **x** |  |
| [55] |  |  |  | **x** | **x** |  |  |  |  |  |  |  |  |  |  |  |  |  |  |  |  |  |  |  |  |  |  |  |  |  |  |  | **x** |  | **x** | **x** |
| [25] |  |  | **x** | **x** |  |  | **x** |  | **x** | **x** |  |  |  |  |  |  |  |  |  | **x** |  | **x** |  |  |  |  |  |  |  |  |  | **x** | **x** |  |  |  |
| [48] |  |  | **x** |  |  |  |  |  |  | **x** |  |  |  | **x** | **x** |  |  |  |  | **x** |  | **x** |  |  | **x** |  |  |  |  |  |  |  |  |  |  |  |
| [27] |  |  |  | **x** |  |  |  |  |  | **x** |  |  |  |  |  |  |  |  |  |  | **x** | **x** | **x** |  |  |  |  |  |  | **x** | **x** |  | **x** | **x** |  |  |
| [34] |  |  |  |  |  | **x** |  |  | **x** |  | **x** |  |  | **x** |  |  |  | **x** | **x** |  | **x** | **x** |  |  |  |  |  |  |  |  | **x** |  |  |  |  |  |
| [28] |  |  | **x** | **x** |  | **x** | **x** |  | **x** | **x** |  |  |  |  |  |  |  |  | **x** |  |  | **x** |  |  |  |  |  |  |  |  | **x** |  | **x** |  |  |  |
| [29] |  | **x** |  | **x** |  | **x** |  |  | **x** | **x** |  |  |  | **x** | **x** | **x** | **x** |  | **x** |  | **x** | **x** | **x** | **x** | **x** |  |  | **x** |  | **x** | **x** |  | **x** |  | **x** |  |
| [30] |  | **x** |  | **x** |  | **x** | **x** |  | **x** |  |  |  |  |  |  |  |  |  |  | **x** |  | **x** |  | **x** |  |  |  | **x** |  |  | **x** | **x** |  |  | **x** | **x** |
| [51] |  | **x** |  |  |  |  |  |  |  |  |  |  |  |  | **x** |  |  |  |  |  |  |  |  |  |  |  |  |  |  |  |  |  | **x** |  | **x** |  |
| [43] |  |  |  |  |  | **x** |  |  |  |  |  |  |  |  | **x** |  |  |  |  |  |  | **x** | **x** |  | **x** |  | **x** |  |  |  |  | **x** | **x** |  | **x** |  |
| [35] |  | **x** |  | **x** |  |  |  |  | **x** | **x** |  |  |  |  |  |  |  |  |  |  | **x** | **x** | **x** | **x** | **x** |  |  |  |  |  |  |  | **x** |  |  | **x** |
| [38] |  | **x** | **x** |  |  |  |  |  |  |  |  | **x** |  |  |  |  | **x** | **x** | **x** | **x** | **x** | **x** |  |  | **x** |  |  |  |  |  |  |  |  |  |  | **x** |
| [31] |  | **x** | **x** |  |  |  | **x** |  | **x** |  |  |  |  |  |  | **x** |  |  |  |  |  | **x** |  | **x** |  |  |  | **x** |  |  |  |  |  |  | **x** |  |
| [52] |  |  |  |  | **x** | **x** |  |  |  |  |  |  |  |  |  |  |  |  |  |  |  |  |  |  |  |  |  | **x** | **x** | **x** | **x** |  | **x** |  | **x** | **x** |
| [56] |  |  |  |  |  |  |  |  |  |  |  |  |  |  |  |  |  |  |  |  |  |  |  |  |  |  |  |  |  |  |  |  |  |  |  |  |
| [53] | **x** | **x** |  | **x** |  | **x** |  |  | **x** | **x** |  |  |  |  | **x** |  |  |  |  | **x** | **x** | **x** |  |  |  |  |  |  |  |  |  |  |  |  |  |  |
| [46] | **x** | **x** |  | **x** |  | **x** |  |  | **x** | **x** |  |  |  |  | **x** |  |  |  |  | **x** | **x** | **x** |  |  |  |  |  |  |  |  |  |  |  |  |  |  |
| [49] |  | **x** | **x** | **x** | **x** | **x** | **x** |  |  |  | **x** |  | **x** |  |  |  |  | **x** | **x** |  |  | **x** |  |  |  |  |  |  |  |  |  |  |  |  |  |  |
| [36] |  |  | **x** | **x** |  | **x** |  |  | **x** | **x** |  |  |  |  |  |  |  |  |  |  | **x** | **x** |  | **x** | **x** |  |  |  |  |  | **x** |  | **x** |  |  |  |
| [47] |  |  | **x** | **x** | **x** |  |  |  | **x** |  |  |  |  | **x** |  |  |  |  |  |  | **x** | **x** | **x** | **x** |  |  |  |  |  |  |  |  |  |  |  |  |

Black areas indicate CCM components not targeted by the intervention. Empty cells indicate that studies included all four chronic care model components but did not report specific subcomponents. * indicates articles with lower methodological quality (Busetto, L., Luijkx, K.G., Elissen, A.M.J., Vrijhoef, H.J.M., unpubl.)
